# Two new species of *Conostigmus* Dahlbom, 1858 (Hymenoptera, Megaspilidae) from Xizang Autonomous Region, China

**DOI:** 10.3897/zookeys.1287.200178

**Published:** 2026-08-07

**Authors:** Yu-fan Liu, Wen-Jing Zhao, Xing Yang, Liu-kang Li, Yi-xin Huang, Xu Wang

**Affiliations:** 1 Anhui Provincial Key Laboratory of the Conservation and Exploitation of Biological Resources, Key Laboratory of Biotic Environment and Ecological Safety in Anhui Province, College of Life Sciences, Anhui Normal University, Wuhu, Anhui 241000, China Anhui Provincial Key Laboratory of the Conservation and Exploitation of Biological Resources, Key Laboratory of Biotic Environment and Ecological Safety in Anhui Province, College of Life Sciences, Anhui Normal University Wuhu China; 2 Collaborative Innovation Center of Recovery and Reconstruction of Degraded Ecosystem in Wanjiang Basin Co-founded by Anhui Province and Ministry of Education, School of Ecology and Environment, Anhui Normal University, Wuhu, Anhui 241000, China Collaborative Innovation Center of Recovery and Reconstruction of Degraded Ecosystem in Wanjiang Basin Co-founded by Anhui Province and Ministry of Education, School of Ecology and Environment, Anhui Normal University Wuhu China

**Keywords:** Ceraphronoidea, DNA barcoding, identification key, morphology, parasitoid wasp, taxonomy

## Abstract

The genus *Conostigmus* Dahlbom, 1858 (Hymenoptera: Megaspilidae) is the most species-rich genus within the family Megaspilidae, with a worldwide distribution and important ecological roles as parasitoids. Based on morphological evidence, two new species of the genus, namely *Conostigmus vitreus***sp. nov**. and *Conostigmus obsidianus***sp. nov**., are described and illustrated from Xizang Autonomous Region, China. An updated key to the Chinese species of *Conostigmus* is provided. This study contributes additional new taxa to the fauna of *Conostigmus* in Xizang, further enriching the knowledge of the Himalayan Megaspilidae fauna.

## Introduction

Megaspilidae Ashmead, 1893 is a globally distributed family of small parasitoid wasps in the superfamily Ceraphronoidea, occurring across all major biogeographic regions except Antarctica (Johnson and Musetti 2004; Mikó et al. 2011; Bijoy et al. 2014; Pezzini and Köhler 2020; Wang et al. 2025). Members of the family can be readily distinguished by a suite of diagnostic morphological characters: a forewing with a well-developed large pterostigma; the radial sector crossvein (R-rs) of the forewing curved; all tibiae with two apical spurs; and a mesoscutum typically with three longitudinal furrows, sometimes reduced. Currently, more than 450 valid species in 12 genera are recognized worldwide, with intensive recent revisions indicating that a large proportion of species diversity remains undescribed (Iemma et al. 2016; Salden and Peters 2023).

The genus *Conostigmus* Dahlbom, 1858 was initially established as a subgenus of *Megaspilus* Westwood, 1833 and was later elevated to generic rank by Kieffer (1907). As the most species-rich and widely distributed genus within Megaspilidae, *Conostigmus* currently includes approximately 190 valid described species, with the highest diversity concentrated in the Palaearctic region (Johnson and Musetti 2004; Trietsch et al. 2020; Cui et al. 2023; Qian et al. 2024; Wang et al. 2024; Yang et al. 2025). In contrast, only 17 species have been formally recorded from China to date (Dessart 1997; Cui et al. 2023; Qian et al. 2024; Wang et al. 2024, 2025; Yang et al. 2025). Diagnostic characters for *Conostigmus* include cylindrical male flagellomeres, female F1 less than 1.5× as long as the pedicel, and OOL equal to or longer than POL (Dessart 1972; Fergusson 1980; Alekseev and Radchenko 2001; Macedo and Kawada 2013; Mikó et al. 2016, 2018; Wang et al. 2025). Strong sexual dimorphism is widespread in *Conostigmus*, often making morphological association of conspecific males and females difficult or unreliable (Yang et al. 2025). For this reason, DNA barcode data, especially sequences of the mitochondrial cytochrome *c* oxidase subunit I (COI) gene, have become increasingly important for confirming conspecificity and linking sexes in taxonomic studies of the genus.

In the present study, two new species of *Conostigmus* collected from the Xizang Autonomous Region, China, *Conostigmus vitreus* Liu & Wang, sp. nov. and *Conostigmus obsidianus* Liu & Wang, sp. nov., are described and illustrated. We also provide COI barcode sequences for both new species, which support morphological species delimitation and facilitate sex association. This work expands the known diversity of Chinese *Conostigmus* and provides new molecular resources for future phylogenetic and biogeographic analyses of Megaspilidae.

## Material and methods

Specimens examined in this study were collected using sweep nets, Malaise traps, and yellow pan traps. All specimens were preserved in absolute ethanol immediately after collection and deposited in the Insect Collection of Anhui Normal University (**AHNU**), Wuhu, China. Specimen accession codes used in this study follow a standardized prefix system: “XZMT-” denotes specimens collected from Medog (Motuo), Xizang (Tibet), and “MXZ-” is the abbreviation for Malaise trap collections from Xizang.

For morphological imaging, specimens were air-dried and stabilized with molding clay before observation. Imaging was performed using a Leica M205A stereomicroscope equipped with a Leica DFC-500 digital camera. Image stacking was processed using the Leica Application Suite (LAS) software, and final image plates were edited in Adobe Photoshop software (CS3 or 2020) for labeling, brightness/contrast adjustment, and sharpening.

For male genitalia dissection, the apical metasomal segments were detached and macerated in 35% H_2_O_2_ in a boiling water bath for 5 min. After cooling, segments were transferred to a drop of glycerin on a concavity slide and dissected using #5 fine forceps and #2 insect pins. Dissected genitalia were stored in glycerin for subsequent morphological examination. All linear measurements are given in micrometers (μm), except for body length (excluding antennae), which is provided in millimeters (mm). Morphological terminology follows the Hymenoptera Anatomy Ontology (Yoder et al. 2010); additional terms and abbreviations follow Mikó and Deans (2009), Trietsch et al. (2020), and Bijoy and Rajmohana (2021) (Table 1).

**Table 1.** Abbreviations and morphological terms used in text.

**Abbreviations**	**Paraphrase**
EHf	Eye height, frontal view
F1, F2, ..., F9	Flagellomere 1, 2, ..., 9
HH	Head height, anterior view
HL	Head length
HW	Head width
IOS	Interorbital space
LOL	Lateral ocellar length, shortest distance between inner margins of median and lateral ocelli
OOL	Ocular ocellar length, minimum distance between a posterior ocellus to the eye margin
POL	Posterior ocellar length, shortest distance between inner margins of posterior ocelli
S9	9^th^ abdominal sternite

Genomic DNA was non-destructively extracted from whole specimens using the TIANamp Genomic DNA Kit. After extraction, all specimens were rinsed three times with absolute ethanol and then returned to absolute ethanol for long-term preservation, ensuring their availability for subsequent morphological examination and as voucher specimens. The COI barcode region was amplified using the universal primers Cer_COI_F/HCO2198 (Folmer et al. 1994). PCR amplifications were performed in 25 µL reactions containing 2 µL DNA template, 8.5 µL ddH_2_O, 12.5 µL PCR master mix, and 2 µL primer mix. Thermal cycling conditions were: initial denaturation at 94 °C for 2 min; 5 cycles of 96 °C for 1 min, 48 °C for 1 min, 72 °C for 2 min 10 s; 35 cycles of 93 °C for 1 min, 53 °C for 1 min, 72 °C for 2 min 10 s; and a final extension at 72 °C for 5 min. All newly generated COI sequences were submitted to GenBank (Table 2).

**Table 2.** Taxa used in the phylogenetic analyses including barcode sequence length and GenBank accession numbers.

**Species**	**Length (bp)**	**Family**	**GenBank accession number**
*Aphanogmus kretschmanni*	637	Ceraphronidae	OP722469
*Aphanogmus fijiensis*	510	Ceraphronidae	GMBCB2052-15 (BOLD Process ID)
*Dendrocerus carpenteri*	578	Megaspilidae	MF154183
*Dendrocerus lui*	566	Megaspilidae	PX935704
*Dendrocerus anisodontus*	566	Megaspilidae	PX935705
*Conostigmus longus*	627	Megaspilidae	OR797514
*Conostigmus asperatus*	627	Megaspilidae	OR797511
*Conostigmus vitreus* Liu & Wang, sp. nov. (XZMT-54)	566	Megaspilidae	PX935707
*Conostigmus vitreus* Liu & Wang, sp. nov. (XZMT-39)	566	Megaspilidae	PX935706
*Conostigmus obsidianus* Liu & Wang, sp. nov. (MXZ-21)	566	Megaspilidae	PX935708

Maximum likelihood (ML) phylogenetic analysis was performed using *Aphanogmus kretschmanni* Moser, Ulmer, Kamp, Vasili, Renninger, Mikó & Krogmann, 2023 and *Aphanogmus fijiensis* (Ferriere, 1933) as outgroups. The dataset comprised two *Conostigmus* and three *Dendrocerus* Ratzeburg, 1852 from this study. Sequence alignment and pairwise genetic distance were conducted in MEGA-X. The ML tree was reconstructed on the IQ-TREE web server with the TIM2+F+I+R2 model selected automatically. The phylogenetic tree was visualized and polished using the TVBOT platform.

## Results

### Molecular analysis

Three COI sequences were newly generated in this study, including two representative sequences of *Conostigmus vitreus* Liu & Wang, sp. nov. and one sequence of *Conostigmus obsidianus* Liu & Wang, sp. nov. In total, 10 COI sequences representing nine species were incorporated into the molecular analysis. Pairwise corrected genetic distances are presented in Table 3.

**Table 3.** Pairwise genetic distances of COI among eight Megaspilidae species.

**Species**	**1**	**2**	**3**	**4**	**5**	**6**	**7**	**8**	**9**	**10**
1. *C. vitreus* Liu & Wang, sp. nov.										
2. *C. vitreus* Liu & Wang, sp. nov.	0.000									
3. *C. obsidianus* Liu & Wang, sp. nov.	0.111	0.111								
4. *C. longus*	0.117	0.117	0.128							
5. *C. asperatus*	0.219	0.219	0.240	0.221						
6. *D. carpenteri*	0.210	0.210	0.198	0.227	0.257					
7. *D. lui*	0.180	0.180	0.187	0.202	0.261	0.156				
8. *D. anisodontus*	0.212	0.212	0.223	0.201	0.275	0.222	0.206			
9. *A. kretschmanni*	0.366	0.366	0.366	0.374	0.408	0.366	0.352	0.422		
10. *A. fijiensis*	0.263	0.263	0.248	0.241	0.329	0.272	0.278	0.338	0.259	

**Figure 1.**
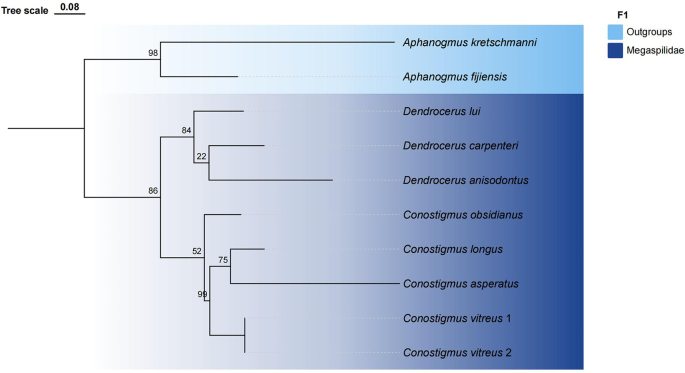
Maximum likelihood (ML) phylogeny of *Conostigmus* based on the barcode region of mitochondrial cytochrome oxidase 1. Bootstrap support values are indicated at internal nodes.

Three specimens of *C. vitreus* Liu & Wang, sp. nov. and three specimens of *C. obsidianus* Liu & Wang, sp. nov. were subjected to DNA barcoding, yielding two valid sequences for *C. vitreus* Liu & Wang, sp. nov. and one valid sequence for *C. obsidianus* Liu & Wang, sp. nov. The intraspecific genetic distance of *C. vitreus* Liu & Wang, sp. nov. was 0%, supporting congruence between molecular species delimitation and morphological taxonomy. Interspecific genetic distances ranged from 11.1% to 25.7%. All species were supported in the maximum likelihood (ML) tree (Fig. 1).

All species were recovered as well-supported monophyletic lineages in the ML tree (Fig. 1). Both new species were placed within the genus *Conostigmus*, which formed a strongly supported monophyletic group with a bootstrap support value of 84%. *Conostigmus vitreus* Liu & Wang, sp. nov. and *Conostigmus obsidianus* Liu & Wang, sp. nov. each formed distinct clades within *Conostigmus*. The two individuals of *C. vitreus* [*C. vitreus* 1 (female) and *C. vitreus* 2 (male)] clustered together with 90% bootstrap support, confirming their conspecificity and providing molecular evidence for the reliable association of males and females of this species.

### Taxonomy

#### Key to the species of *Conostigmus* from China

**Table:** 

1	Mesosoma length > 2.0× maximum width; preoccipital furrow absent	***Conostigmus ampullaceus* Dessart, 1997**
–	Mesosoma length ≤ 2.0× maximum width	**2**
2	Facial pit absent	**3**
–	Facial pit present	**7**
3	Anteromedian projection of the metanoto-propodeo-metapecto-mesopectal complex present	**4**
–	Anteromedian projection of the metanoto-propodeo-metapecto-mesopectal complex absent	**5**
4	Sternaulus reaching about 2/3 length of mesopleuron	**6**
–	Sternaulus reaching about 4/5 length of mesopleuron	***Conostigmus clayulatus* Yang & Wang, 2025**
5	Harpe shorter than gonostipes	***Conostigmus nankunensis* Qian & Wang, 2024**
–	Harpe longer than gonostipes	***Conostigmus abdominalis* Boheman, 1832**
6	Syntergum smooth, longer than wide	***Conostigmus obsidianus* Liu & Wang, sp. nov**.
–	Syntergum smooth, wider than long	***Conostigmus asperatus* Wang & Zhu, 2025**
7	Flagellomere 1 (F1) longer than scape	***Conostigmus longus* Wang & Zhu, 2025**
–	Flagellomere 1 (F1) shorter than or equal in length to scape	**8**
8	Body length (excluding antennae) ≥ 2.0 mm	**9**
–	Body length (excluding antennae) < 2.0 mm	**11**
9	Basal gastral carinae length ≥ 1/3 of syntergum length	**10**
–	Basal gastral carinae length < 1/3 of syntergum length; harpe longer than gonostipes	***Conostigmus xui* Cui & Wang, 2023**
10	F1 subequal to scape; head and mesosoma reddish brown	***Conostigmus electrinus* Wang & Chen, 2024**
–	F1 shorter than scape; head and mesosoma black	***Conostigmus villosus* Dessart, 1997**
11	Preoccipital furrow present and extends into the ocellar triangle	**12**
–	Preoccipital furrow present but does not extend into the ocellar triangle	**15**
12	Harpe conical with a pointed apex	***Conostigmus acutus* Wang & Chen, 2024**
–	Harpe strip-shaped without a pointed apex	**13**
13	Gastral carinae length > 1/5 of syntergum length	**14**
–	Gastral carinae length < 1/5 of syntergum length	***Conostigmus bomensis* Wang & Zhu, 2025**
14	Total length of male genitalia < 130 μm, highly translucent and hyaline in appearance	***Conostigmus vitreus* Liu & Wang, sp. nov**.
–	Total length of male genitalia ≥ 130 μm, brownish and sclerotized	***Conostigmus jilongensis* Wang & Zhu, 2025**
15	Mesometapleural sulcus continuously concave	**16**
–	Mesometapleural sulcus smooth and linear	**18**
16	Harpe shorter than gonostipes in lateral view, reaching 3/5 of gonostipes	***Conostigmus rufinotum* Dodd, 1914**
–	Harpe slightly longer than gonostipes	**17**
17	Scutellum as long as wide; dorsal margin of S9 straight	***Conostigmus rotundus* Yang & Wang, 2025**
–	Scutellum longer than wide; dorsal margin of S9 protruded	***Conostigmus quadripetalus* Wang & Chen, 2024**
18	Sternaulus complete, pterostigma 3.6× as long as wide	***Conostigmus kairus* Wang & Zhu, 2025**
–	Sternaulus incomplete, pterostigma 3.2× as long as wide	***Conostigmus shigatsensis* Wang & Zhu, 2025**

##### Conostigmus vitreus

Taxon classificationAnimaliaHymenopteraMegaspilidae

Liu & Wang sp. nov.

82A02959-5CF3-5F23-9225-5219A32F7E4B

https://zoobank.org/5F9CC2BE-96DB-43EA-986A-48D2B4A88FEF

Figs 2, 3, 4

###### Diagnosis.

Male genitalia small and highly translucent; harpe shorter than gonostipes, with numerous slender, distally oriented apical setae; parossiculus not medially fused; gonostipes longer than wide, not fused with parossiculi.

###### Material examined.

***Holotype***. China • ♂, Xizang Autonomous Region, Nyingchi City, Medog County, Damu Township, 29.50809°N, 95.47287°E, alt. 1814 m, 11 May 2017; XZMT-44 (AHNU). ***Paratypes***. China • 2♂♂1♀, same data as holotype, XZMT-39/53/54 (AHNU).

###### Description.

**Male**: Body length 1.1–1.5 mm (*N* = 3).

**Figure 2.**
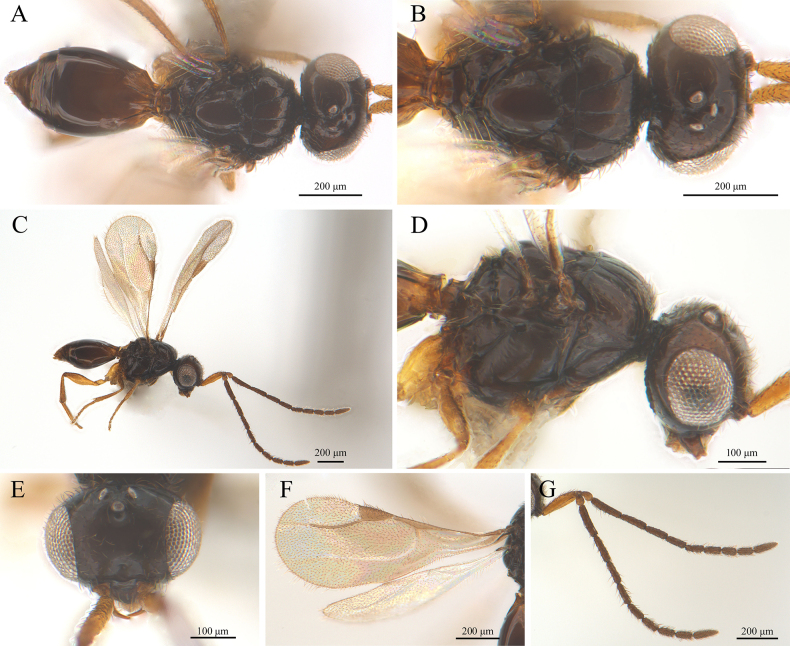
*Conostigmus vitreus* Liu & Wang, sp. nov., male, holotype. **A**. Dorsal habitus; **B**. Head and mesosoma, dorsal view; **C**. Lateral habitus; **D**. Head and mesosoma, lateral view; **E**. Head, anterior view; **F**. Wings; **G**. Antennae, lateral view.

***Coloration***. (Fig. 2) Head, mesosoma, and metasoma mostly black. Antennal scape light brown, pedicel dark brown, flagellomeres black. Ocelli and compound eyes silvery. Legs light brown. Pterostigma, costa, and radial vein pale brown. Body with short, pale yellowish-brown pubescence; wing marginal cilia light brown.

***Head***. (Fig. 2B, D, E) Head approximately 1.3× as wide as mesosoma. HH: EHf = 1.7–1.9; HH: HL = 1.3–1.4; HW: IOS = 1.6–1.7; HW: HH = 1.1–1.5. OOL longer than POL, ocellar triangle with narrow base; OOL: LOL = 2.6–3.5; POL: OOL = 0.5–0.6. Head oval, covered with short pubescence. Intertorular carina present; facial pit present; preoccipital furrow present; preocellar pit present; facial sulcus absent.

***Antennae***. (Fig. 2C) Scape length: pedicel length = 3.7–5.1; pedicel small, nearly teardrop-shaped. Scape length: F1 length = 1.3–1.7; F1 length: F2 length = 1.1–1.2; F1 length: pedicel length = 2.7–3.1. F1 longest, followed by F9. F7 short; F4 and F5 subequal in length.

***Mesosoma***. (Fig. 2B, D) Mesosoma slender, 1.5× as long as wide (length/width/height = 498/324/351 μm), densely pubescent with granular surface sculpture. Mesoscutum 1.9× as wide as long (length/width = 167/324 μm). Transscutal articulation distinct, separating mesonotum from scutello-axillar complex. Notaulus complete, posterior end adjacent to transscutal articulation. Median mesoscutal sulcus present, posterior end adjacent to transscutal articulation. Scutellum 1.1× as long as wide, delimited by U-shaped carina. Sternaulus present; dorsal mesometapleural carina present; anterior mesopleural sulcus present; antemedian projection of metanoto-propodeo-metapecto-mesopectal complex present.

***Wings***. (Fig. 2F) Fore wing 1.1–1.4 mm long, well developed, apex extending past petiole, hyaline with weak dark pigmentation, pterostigma nearly triangular, length: width = 2.4. Radius (250 μm) weakly curved medially, 1.5× as long as pterostigma length. Hind wing venation reduced, hyaline.

***Metasoma***. (Fig. 2A, G) Metasoma 2.0× as long as wide (length/width/height = 576/288/242 μm). Syntergum smooth, longer than wide, with three distinct gastral carinae, approximately 0.4× as long as syntergum length. Syntergal translucent patch present, transverse and irregularly cylindrical. Remaining tergites smooth, with sparse lateral setae.

**Figure 3.**
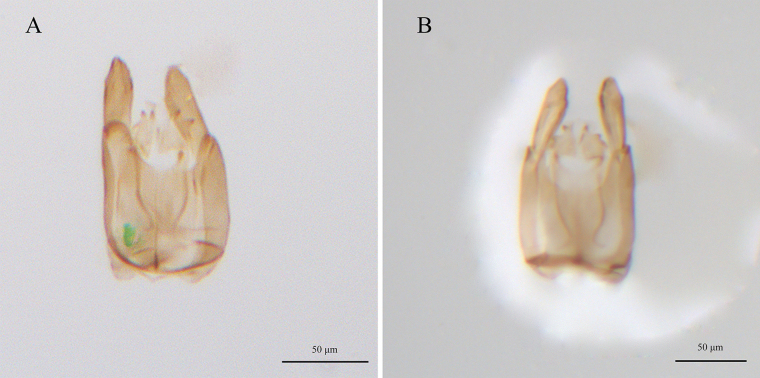
*Conostigmus vitreus* Liu & Wang, sp. nov., male, holotype, genitalia. **A**. Dorsal view; **B**. Ventral view.

***Male genitalia***. (Fig. 3) Small (length/width = 125/70 μm), highly translucent. Harpe shorter than gonostipes, simple (not bilobed), medially oriented with numerous slender apical setae directed distally. Parossiculi not medially fused. Gonostipes longer than wide, not fused with parossiculi.

**Figure 4.**
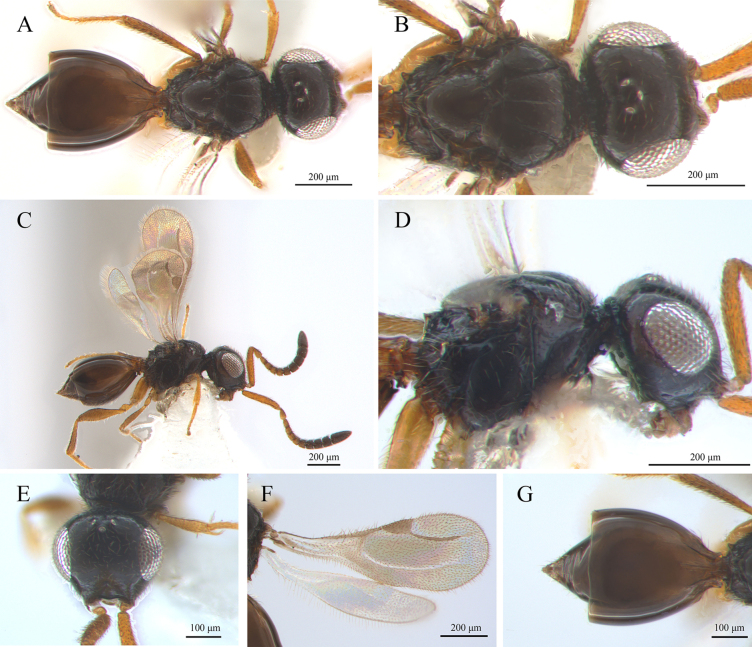
*Conostigmus vitreus* Liu & Wang, sp. nov., female, paratype. **A**. Dorsal habitus; **B**. Head and mesosoma, dorsal view; **C**. Lateral habitus; **D**. Head and mesosoma, lateral view; **E**. Head, anterior view; **F**. Wings; **G**. Metasoma, dorsal view.

**Female**: (Fig. 4) Similar to male, except as follows: body length 1.2 mm (male 1.3–1.5 mm); metasoma blackish-brown (male black); scape, pedicel, and F1–F4 orange-yellow (male scape light brown, pedicel dark brown, flagellomeres black); F5–F9 brown (male black); antennal pedicel long, slightly longer than F1–F8 (male pedicel small, teardrop-shaped, distinctly shorter).

###### Remarks.

This species is similar to *Conostigmus jilongensis* Wang & Zhu, 2025, but can be distinguished by the following characters: male genitalia small and highly translucent (vs. large and sclerotized, brown in *C. jilongensis*).

###### Distribution.

China (Xizang).

###### Etymology.

Consistent with the genus name, the species name is a Latin masculine adjective meaning “glassy” or “transparent”, referring to the small, vitreous male genitalia in this species.

##### Conostigmus obsidianus

Taxon classificationAnimaliaHymenopteraMegaspilidae

Liu & Wang sp. nov.

876A5043-EA67-539E-BB68-E1E40F4DE368

https://zoobank.org/CAB46079-1F74-45C8-A0B9-8A561AB8BBF0

Figs 5, 6, 7

###### Diagnosis.

Body entirely pitch-black, including head, mesosoma, metasoma, antennae, and legs; wings hyaline with dark brown pterostigma; male genitalia large and strongly pigmented; harpe broad basally, tapering to an acute, leaf-like apex, with setae concentrated along lateral margin and longer curved apical setae; aedeagus short, concealed within harpe with only apex exposed.

###### Material examined.

***Holotype***. China • ♂; Xizang Autonomous Region, Gyirong County, 28.5189°N, 85.2181°E, alt. 3354 m, 23 July 2023; MXZ-18 (AHNU). ***Paratypes***. China • 2♀♀, same data as holotype, MXZ-1/21 (AHNU).

###### Description.

**Male**: Body length: 1.8 mm (*N* = 1).

**Figure 5.**
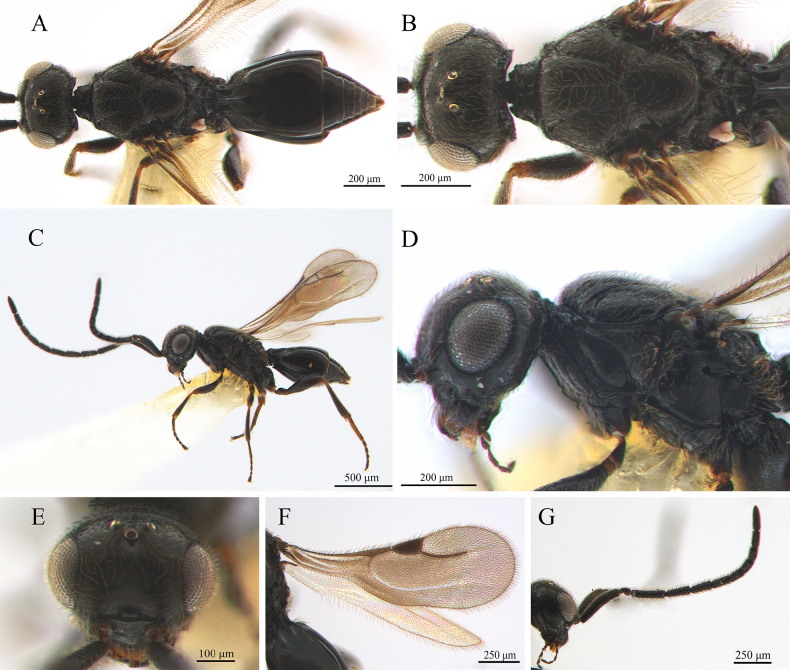
*Conostigmus obsidianus* Liu & Wang, sp. nov., male, holotype. **A**. Dorsal habitus; **B**. Head and mesosoma, dorsal view; **C**. Lateral habitus; **D**. Head and mesosoma, lateral view; **E**. Head, anterior view; **F**. Wings; **G**. Antennae, lateral view.

***Coloration***. (Fig. 5) Body uniformly black; only wings hyaline and translucent. Head entirely shiny black; compound eyes black, smooth and glossy. Antenna entirely black, without contrasting markings. Mesosoma and metasoma uniformly black, abdominal segment junctions without pale color transitions. Legs entirely black. Fore and hind wings hyaline and membranous; veins pale brown to yellowish-brown; pterostigma dark brown.

***Head***. (Fig. 5B, D, E) As wide as the mesosoma. HH: EHf = 2.2; HH: HL = 1.4; HW: IOS = 1.6; HW: HH = 1.0. OOL longer than POL, ocellar triangle with narrow base; OOL: LOL = 2.7. POL: OOL = 0.8. Head oval, covered with short pubescence. Intertorular carina present; facial pit absent; preoccipital furrow present.

***Antennae***. (Fig. 5C, G) Scape distinctly longer than pedicel, stout and rod-shaped, slightly tapering apically. Pedicel teardrop-shaped, shortest antennal. Scape length: pedicel length = 4.5. F1 longest, F1 length: F5 length = 2.7, with marked length gradient. Flagellomeres shortening from F1 to F5, then lengthening from F5 to F9; F9 longest apical flagellomere.

***Mesosoma***. (Fig. 5B, D) Mesosoma slender and depressed, 1.5× as long as wide (length/width/height = 674/440/394 μm). Mesoscutum 1.9× as wide as long (length/width = 228/440 μm), broad and convex, finely reticulate-punctate, sparsely covered with long, curved, posteriorly directed setae. Transscutal articulation distinct, separating mesonotum from scutello-axillar complex. Notaulus complete, posterior end adjacent to transscutal articulation. Median mesoscutal sulcus present, posterior end adjacent to transscutal articulation. Scutellum 1.2× as long as wide, delimited by U-shaped carina. Scutoscutellar sulcus present, medially angled and foveolate. Anterior mesopleural area finely reticulate-punctate, separated from posterolateral region by anterior mesopleural sulcus. Antemedian projection of metanoto-propodeo-metapecto-mesopectal complex present.

***Wings***. (Fig. 5F) Fore wing 1.4 mm long, well-developed, narrow-elongate, apex extending past posterior margin of petiole, hyaline with weak dark pigmentation; wing margin with fine short cilia. Pterostigma nearly triangular, length: width = 2.3. Radius (282 μm) weakly curved medially, 1.5× as long as pterostigma length. Hind wing venation reduced, hyaline.

***Metasoma***. (Fig. 5A, C) Metasoma 1.7× as long as wide (length/width/height = 824/481/373 μm). Syntergum smooth, longer than wide, with three distinct gastral carinae. Syntergal translucent patch present, transverse and irregularly cylindrical. Remaining tergites smooth, with sparse lateral setae.

**Figure 6.**
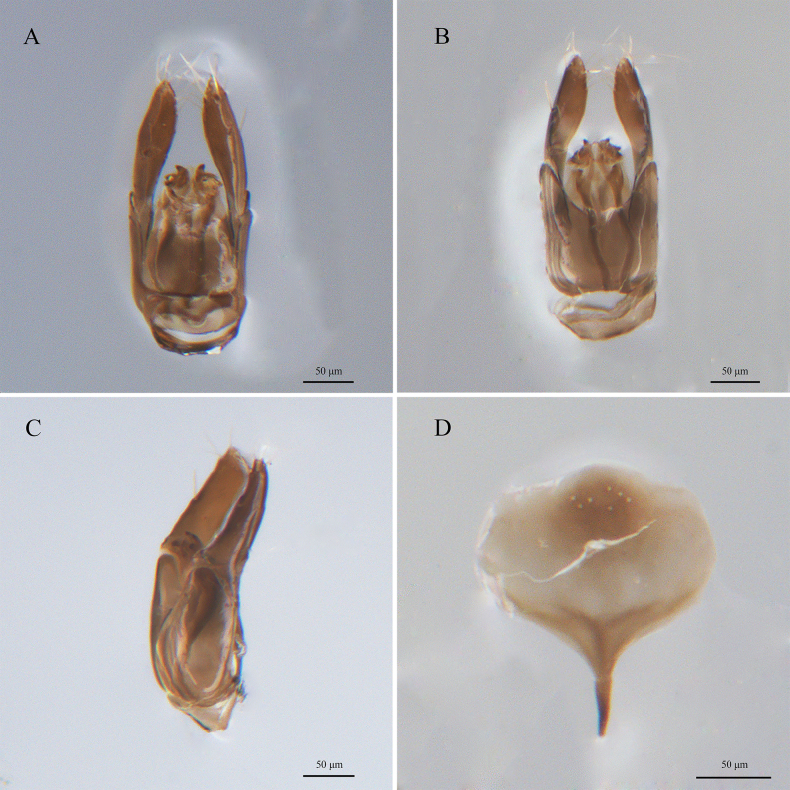
*Conostigmus obsidianus* Liu & Wang, sp. nov., male, holotype, genitalia. **A**. Dorsal view; **B**. Ventral view; **C**. Lateral view; **D**. S9.

***Male genitalia***. (Fig. 6) Genitalia symmetrical, elongate-elliptical, large, strongly sclerotized, dark brown. Harpe broad basally, tapering to an acute, leaf-like apex; each harpe with 4–6 elongate apical setae; harpe simple, medially oriented; inner margin with small tooth-like projections, curved dorsally. Parossiculi not medially fused. Aedeagus short, concealed within harpe, only apex exposed. S9 broadly trapezoidal, transverse width greater than median length; anterior margin broadly rounded, with small semicircular projections and six setal patches.

**Figure 7.**
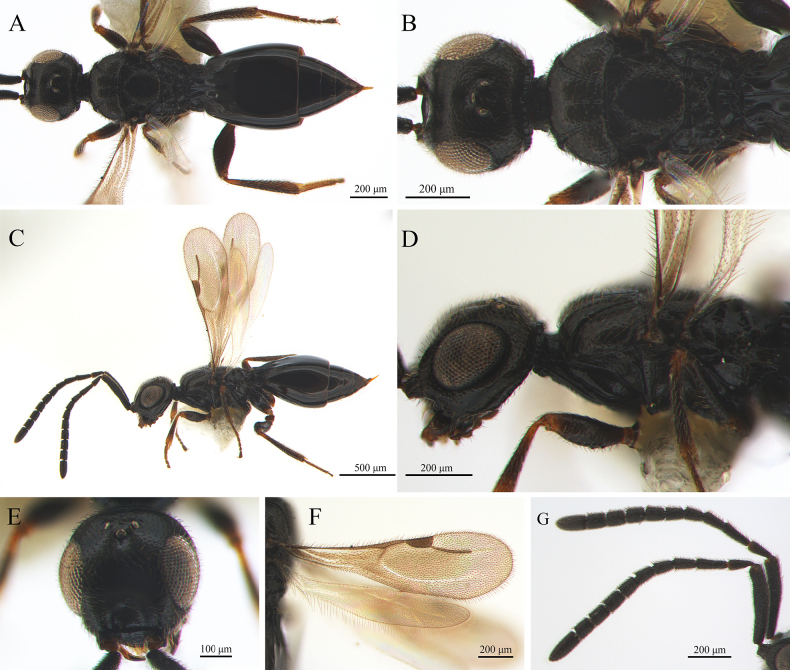
*Conostigmus obsidianus* Liu & Wang, sp. nov., female, paratype. **A**. Dorsal habitus; **B**. Head and mesosoma, dorsal view; **C**. Lateral habitus; **D**. Head and mesosoma, lateral view; **E**. Head, anterior view; **F**. Wings; **G**. Antennae, lateral view.

**Female**: (Fig. 7) Similar to male except as follows: body length 1.7–2.2 mm (male 1.8 mm); antennal pedicel long, slightly longer than flagellomeres F2–F8 (male pedicel small, teardrop-shaped, distinctly shorter than any flagellomere).

###### Remarks.

This species is similar to *Conostigmus asperatus* Wang & Zhu, 2025, but can be distinguished by its uniformly pitch-black body (vs. head and mesosoma black, metasoma dark brown, scape and pedicel yellow in *C. asperatus*), head as wide as mesosoma (vs. slightly wider), syntergum longer than wide (vs. wider than long), and male genitalia large, dark brown, strongly sclerotized, with harpe broad basally and tapering to a leaf-like apex (vs. brownish-yellow, weakly sclerotized, harpe simple and apically narrowed).

###### Distribution.

China (Xizang).

###### Etymology.

Consistent with the genus name, the species name is a Latin masculine adjective meaning “obsidian-like” referring to the entirely pitch-black body coloration of this species.

## Supplementary Material

XML Treatment for Conostigmus vitreus

XML Treatment for Conostigmus obsidianus

## Additional information

### Artificial Intelligence (AI) use

The authors accept full responsibility for the content of the manuscript, including the disclosure of any use of AI.

No AI tools were used in the preparation of this manuscript.

### Author ORCIDs

Y.-f. Liu https://orcid.org/0009-0002-5909-1376

W.-J. Zhao https://orcid.org/0009-0009-8199-8442

X. Yang https://orcid.org/0009-0006-0732-0121

L.-k. Li https://orcid.org/0009-0007-1195-5151

Y.-x. Huang https://orcid.org/0000-0002-7885-321X

X. Wang https://orcid.org/0000-0001-6465-4052

### Data availability

All of the data that support the findings of this study are available in the main text.
